# D-Arabinose Methabolism: Characterization of Bifunctional Arabinokinase/Pyrophosphorylase of Leishmania major

**Published:** 2009-10

**Authors:** N.M. Novozhilova, N.V. Bovin

**Affiliations:** 1 Shemyakin and Ovchinnikov Institute of Bioorganic Chemistry, RAS

## Abstract

In this work we describe an unusual enzyme from Leishmania major (Arabinokinase/Pyrophosphorylase) that catalyzes the synthesis of GDP-D-arabinopyranose (GDP-D-Arap) via a D-arabinose-1-phosphate intermediate in the presence of ATP and GTP. Our data indicate GDP-D-Arap transport in vivo by the LPG2 multispecific nucleotide sugar transporter into the Leishmania Golgi apparatus, in which it can be used by glycosyltransferases as a donor substrate for glycosylation.

## Introduction

It is known that both bacteria (Bacteriodes) and plants (Arabidopsis) can synthesize GDP-L-Fuc from L-fucose (L-Fuc) via intermediate L-fucose-1-phosphate using a bifunctional enzyme L-fucokinase/GDP-L-fucose pyrophosphorylase [3, 4]. Since D-Arap and L-Fuc are structurally similar, it makes sense to assume that biosynthesis of GDP-D-Arap in Leishmania can occur through a mechanism similar to that of GDP-L-Fuc biosynthesis in other species. To check this hypothesis, the L.major genome was evaluated for open reading frames homologous to fucokinase and GDP-L-fucose pyrophosphorylase gene sequences. As a result, two near-identical genes (lmjF16.0440 and lmjF16.0480) were found as possessing high homology with the Bacteriodes fragilis fkp and Arabidopsis thaliana at1g01220 genes, both encoding L-fucokinase/GDP-L-fucose pyrophosphorylase [3, 4].

The open reading frames lmjF16.0440 and lmjF16.0480 correspond to putative polypeptides composed of 1,187 aminoacid residues with a calculated molecular mass of 126.5 kDa. The only difference (three aminoacid residues) between these two proteins is the segment 196-199, namely PheGlnAsnHis in LmjF16.0480 and LeuGlnAspTyr in LmjF16.0440. The N-terminal sequences of these proteins contain a highly conserved segment Val100-Lys117 that closely resembles the conserved pyrophosphorylase motif Lys(X)2GlyXThrXMet(X)4Lys [5]. The C-termini also contain a conserved segment Gly950-Ile958 homologous to the GlyXGly(X)2Gly(Ser)2Gly motif forming the ATP-binding pocket of many kinases [6]. The presence of these conserved sequences has allowed to attribute kinase and pyrophosphorylase activities to the proteins' C- and N-ends, respectively.

## Results

**Fig. 1.**
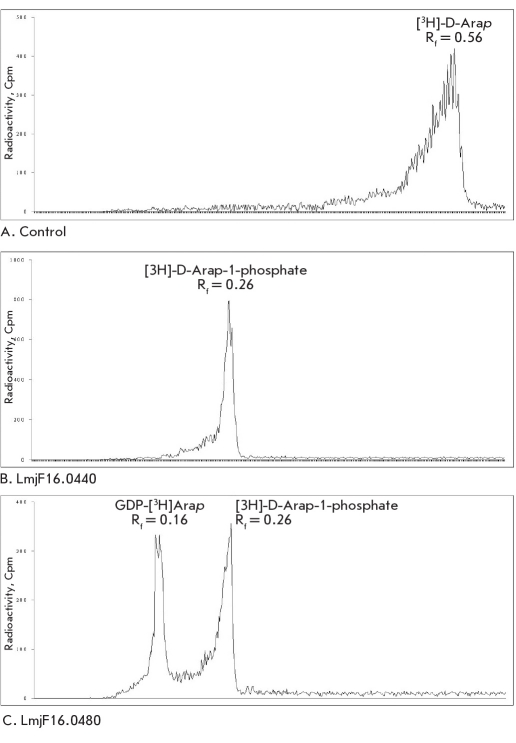
Chromatographic analysis of enzymatic reaction products. Cultures of E. coli BL21(DE3) were transformed with pET16b- LmjF16.0440(His)6 and pET16b- LmjF16.0480(His)6 plasmids and induced with IPTG. Following lysis of the induced cells, the proteins were solubilized with 0.1% Tween 20 and immobilized to a column of TALON resin via N-terminal His-tag. Enzymatic activities of the TALON-bound recombinant LmjF16.0440 and LmjF16.0480 then were tested in vitro. Reaction mixtures contained 5 mM ATP, 5 mM GTP, 5 mM MgSO4 and 0.15 µCi of [3H]D-Arap as substrate. Assay tubes were incubated overnight at 37oC, and the reaction products were analysed by TLC on glass-backed Silica Gel 60 TLC sheets (1-butanol/acidic acid/water, 2:1:1). [3H]D-Arap-containing products were located using a Bio Scan System 200 radiochromatogram scanner. Control assay containing only nonspecific proteins from E. coli cells failed to produce any product (A). Reaction containing the LmjF16.0440 protein produced only D-Arap-phosphate (B), whereas the LmjF16.0480 protein produced [3H]arabinose 1-phosphate and GDP-D-[3H]Arap (C)

The lmjF16.0440 and lmjF16.0480 genes were cloned in the pET16b plasmid vector for further examination of the activities of the putative enzymes. The recombinant LmjF16.0440 and LmjF16.0480 were expressed in E.coli BL21 (DE3)-RIPL cells and tested for enzyme activity (data are shown on Fig. 1). LmjF16.0480 catalyzed the formation of D-Arap-phosphate and GDP-D-Arap, whereas LmjF16.0440 only synthesized D-Arap-phosphate. Reaction products were not observed when the reaction mixture contained only nonspecific proteins extracted from the E.coli cells transformed with an empty pET16b vector. This information allows to conclude that the product of the lmjF16.0480 gene really acts as a bifunctional arabinokinase/pyrophosphorylase; that is, it exhibits both D-arabinokinase and GDP-D-Arap-pyrophosphorylase activity, whereas the recombinant LmjF16.0440 protein only exhibits D-arabinokinase activity.

The necessity for the presence of ATP for kinase activity and GTP for pyrophosphorylase activity in the proteins studied was confirmed when carrying out the enzymatic reactions in the presence of only one triphosphate. Formation of the reaction product was not observed in the absence of both ATP and GTP. When only ATP was present, the reaction was not completed and led to the formation of arabinose-phosphate only. When only GTP was present in the reaction mixture, a small amount of GDP-D-Arap was produced, apparently due to the enzyme's capability to utilize GTP instead of ATP. However, in this case the reaction proceeded slowly with a low yield of the reaction products as compared with the reaction in the presence of both ATP and GTP. Thus, the entire reaction requires both triphosphates in the reaction mixture: ATP - for kinase activity and GTP - for pyrophosphorylase activity of the bifunctional enzymes under study.

The kinetic parameters, namely, the Michaelis constant (Km) and maximum reaction velocity (Vmax), were determined for recombinant LmjF16.0440 and LmjF16.0480. These parameters are shown in Table 1 for each reaction catalyzed by each of the enzymes. Both enzymes demonstrate similar features as kinases. However, an extremely high Km = 67 mM was determined for pyrophosphorylation catalyzed by LmjF16.0480. Such low affinity to arabinose-phophate could suggest the association of both catalytic centers of the enzyme, so that the D-Arap-1-phosphate from the kinase center moves straight to the pyrophosphorylase one. So, a considerable amount of exogenous arabinose-phosphate is necessary for its appearance in the pyrophosphatase active center, which is reflected in the high Km value.

**Table 1:** Kinetic Parameters for Recombinant LmjF16.0440 and LmjF16.0480 Proteins

Enzyme	Substrate	K_m_ (mM)	V_max_ (μM/L/min)
LmjF16.0440	D-Arabinose D-Arabinose-1-phosphate	1.23 -	0.35 -
LmjF16.0480	D-Arabinose D-Arabinose-1-phosphate	2.90 67.0	0.50 160.0

The human genome contains genes that are partially homologous to the bifunctional enzyme from Leishmania. In particular, the locus NP 659496 encodes a protein composed of 1,185 aminoacid residues with 44% homology with the kinase C-end, whereas its N-end has no significant resemblance with pyrophosphorylase domains. The gene was cloned, and its recombinant product was tested for GDP-D-Arap synthesizing capability. This test has shown no activity. The absence of pyrophosphorylase activity for the human protein is not surprising considering the low homology of the human protein N-end with the pyrophosphorylase domains. The D-arabinose-activating capability was also lacking, despite the close resemblance between the kinase C-end and both LmjF16.0440 and LmjF16.0480.

## Conclusion

**Fig. 2.**
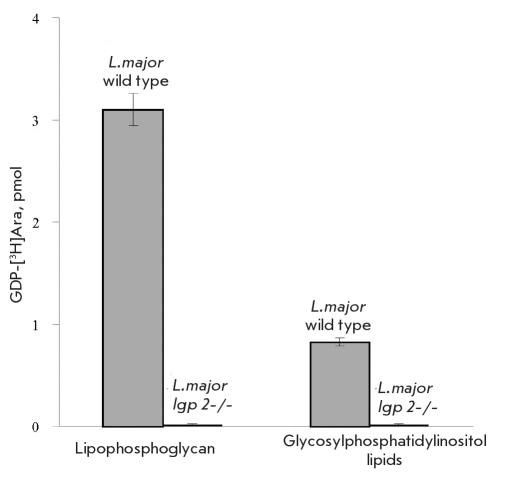
Schematic representation of the system. An elastic plate of length *L*, width *l*, and thickness *h* ≤ *l*, *L* clamped at the origin is embedded in a three-dimensional parallel flow of an inviscid fluid with velocity *U* in the *x* direction. Its transverse position is denoted by *Y* (*x*, *t*). The incomplete cylinders depict the real vortex that is shed from the trailing edge, and an imaginary vortex in the interior of the plate that moves inwards, and is necessary to preserve the impenetrability of the boundary of the plate.

In most cases, activated carbohydrates are synthesized in cytoplasm and subsequently transported into the Golgi lumen, in which they are utilized by corresponding glycosyltransferases as substrate donors in glycosylation reactions. Earlier, the LPG2 protein found in the Leishmania Golgi apparatus membrane was classified as a multispecific transporter that can transport not only GDP-Man, but also GDP-D-Arap and GDP-L-Fuc [7]. To make sure that LPG2 is the only transporter responsible for GDP-D-Arap transport into the Golgi apparatus, we grew the cells of wild-type L. major and its lpg2 knockout mutant (L. major lpg2-/-) in the presence of [3H]-arabinose and determined [3H]Ara incorporated into the cell surface glycoconjugates (fig. 2). One can see from the presented data that the lpg2 knockout cells do not incorporate [3H]-arabinose. The absence of arabinose in lipophosphoglycan of L. major lpg2-/- is easy to explain, because the loss of LPG2 leads to cancellation of GDP-Man transport into the Golgi apparatus with the corresponding termination of the synthesis of the carbohydrate moiety of the molecule [9]. Thus, even in the presence of GDP-Arap transported into the Golgi lumen by another protein, arabinosyltransferases had no acceptor site for arabinose transfer to lipophosphoglycan. There is no telling this about glycosylinositolphospholipids. Glycosylation of these molecules occurs through another biosynthetic pathway with dolichol-phosphate mannose as a donor [10], so the absence of arabinose residues in glycosylinositolphospholipid molecules of L. major lpg2-/- can be explained by cancellation of GDP-Arap transport into the Golgi apparatus in the cells devoid of lpg2 gene.

Thus, our data very likely suggest GDP-D-Arap transport from cytoplasm (in which it is synthesized) into the Golgi lumen by the multi-functional transporter protein LPG2. We have also classified the L. major lmjF16.0480 gene product as a bifunctional arabinokinase/pyrophosphorylase that can synthesize GDP-D-Arap from D-Arap via the intermediate D-arabino-1-phosphate in the presence of both ATP and GTP. 

## Acknowledgements

The study was supported by the Program of Presidium of Russian Academy of Sciences "Molecular and Cell Biology." The authors are grateful to Prof. H. Guo (University of Washington, USA) for assistance in identification and cloning of Leishmania major and human genes and to Prof. S. Turco (University of Kentucky, USA) for fruitful discussions.
